# Mechanisms for lyssavirus persistence in non-synanthropic bats in Europe: insights from a modeling study

**DOI:** 10.1038/s41598-018-36485-y

**Published:** 2019-01-24

**Authors:** Davide Colombi, Jordi Serra-Cobo, Raphaëlle Métras, Andrea Apolloni, Chiara Poletto, Marc López-Roig, Hervé Bourhy, Vittoria Colizza

**Affiliations:** 1 Computational Epidemiology Laboratory, Institute for Scientific Interchange (ISI), Turin, Italy; 2 Physics Department and INFN, University of Turin, via P. Giuria 1, 10125 Turin, Italy; 3 Institut de Recerca de la Biodiversitat, Departament de Biologia Evolutiva, Ecologia i Ciències Ambientals, Universitat de Barcelona, Barcelona, Spain; 4 CIRAD, UMR ASTRE, F-34398 Montpellier, France; 5 ASTRE, Univ Montpellier, CIRAD, INRA, Montpellier, France; 6 Institut Sénégalais de Recherches Agricoles, Laboratoire National de l’Elevage et de Recherche Vétérinaire, Parc Scientifique de Hann, Dakar, Senegal; 7 INSERM, Sorbonne Université, Institut Pierre Louis d’Epidémiologie et de Santé Publique IPLESP, F75012 Paris, France; 8 Institut Pasteur, Unit lyssavirus dynamics and host adaptation, Paris, France

## Abstract

Bats are natural reservoirs of the largest proportion of viral zoonoses among mammals, thus understanding the conditions for pathogen persistence in bats is essential to reduce human risk. Focusing on the European Bat Lyssavirus subtype 1 (EBLV-1), causing rabies disease, we develop a data-driven spatially explicit metapopulation model to investigate EBLV-1 persistence in *Myotis myotis* and *Miniopterus schreibersii* bat species in Catalonia. We find that persistence relies on host spatial structure through the migratory nature of *M. schreibersii*, on cross-species mixing with *M. myotis*, and on survival of infected animals followed by temporary immunity. The virus would not persist in the single colony of *M. myotis*. Our study provides for the first time epidemiological estimates for EBLV-1 progression in *M. schreibersii*. Our approach can be readily adapted to other zoonoses of public health concern where long-range migration and habitat sharing may play an important role.

## Introduction

Bats are reservoir hosts of a large number of zoonotic viral infections, including some of the recently emerged severe infectious diseases affecting humans^1–4^. They are implicated as reservoirs and vectors for transmission of severe acute respiratory syndrome (SARS) and Middle East respiratory syndrome (MERS) coronaviruses, Ebola and Marburg filoviruses, lyssaviruses, Hendra and Nipah viruses^1,2,4^. Recently a novel lineage of influenza A virus was discovered in bats in Central America^5^. A comprehensive analysis of known mammal-virus associations determined that bats harbor a significantly larger proportion of zoonoses compared to all other mammalian orders^6^. Pathogen spillovers from bats to domestic animals and humans may thus represent a serious threat to public health, biodiversity and global security^7^. For this reason, considerable scientific and public health interest is invested into identifying the mechanisms and conditions for cross-species transmission leading to a potential pandemic episode^8,9^. A key step to reach this goal and reduce the risk for humans from pathogens of bat-origin requires the understanding of how bat ecology may influence the infectious disease dynamics, and thus elucidating those aspects that are most critical to the circulation of viruses in bats.

A number of studies have proposed that bats have unique ecological, social, and immunological traits increasing their potential to host zoonotic viruses, however given the large variety of host and pathogen species and the paucity of wildlife data important gaps still remain^2,10^. Here we focus on lyssaviruses (*Rhabdoviridae* family, *Lyssavirus* genus), the agents of rabies diseases. Bats are considered to be the ancestral hosts of lyssaviruses, before the viruses progressively diverged from this common ancestor to many recipient host species^11–13^. To date, bats were found to serve as reservoirs of 15 of the 17 lyssavirus species currently known^14,15^. In Europe, four different lyssavirus species have been isolated in bats, namely *European bat lyssavirus* types 1 and 2 (EBLV-1 and EBLV-2, respectively), *Bokeloh bat lyssavirus* (BBLV), *West Caucasian bat virus* (WCBV) and one tentative species, *Lleida bat lyssavirus*^13,16–19^. Reported in Europe for the first time in 1954^20^, EBLV-1 is the most commonly found species in the continent with a wide distribution across Germany, the Netherlands, Denmark, France, Spain^16,20–24^. It has the potential to cross the species barrier and infect other domestic and wild mammals^20,22,25,26^, as well as humans^19,24,27^, where it causes fatal encephalitis, indistinguishable from other lyssaviruses.

Relatively little is known about the dynamics of EBLV-1 infection in European bats and the conditions for viral transmission and maintenance. Routine programs of passive surveillance were established in Europe at the end of the 80′s to study the distribution, abundance, and epidemiology of lyssavirus infections in bats. The vast majority of EBLV-1 positive cases was found to be associated with the Serotine bat (*Eptesicus serotinus*)^19,20,28^, a largely diffused species in the Eurasian region that almost exclusively roost in buildings close to suburban areas, i.e. displaying a synanthropic behavior. Retrospective investigations of passive surveillance data and reports from active surveillance studies provided however evidence of EBLV-1 circulation (through neutralizing antibodies, viral RNAs) in other bat species^20,29–35^, including *Myotis myotis* in France^30^, Belgium^32^ and Spain^33–35^, and *Miniopterus schreibersii* in France^30^ and Spain^33–35^.

*M. schreibersii* may be particularly important for the spatial diffusion and maintenance of EBLV-1 in European bats. Sequence analyses of EBLV-1 genomes from nine European countries indeed uncovered the geographic separation between phylogeographical clusters of EBLV-1 variants that cannot be fully explained by the geographic distribution of *E. serotinus*^36^, a sedentary bat species^37^. Other bat species are thought therefore to be implicated in EBLV-1 circulation, with migratory species potentially assuming a prominent role in carrying the pathogen across different host populations at distant areas^38^.

In addition to high mobility, the social nature of bats and their colonial aggregation constitute ideal drivers for viral exchange and dispersal^39^. Colony size and species richness were found to be associated with an increased EBLV-1 seroprevalence in Spanish bats^33,35^. Population density and proximity of bats in the same cave provide indeed higher chances for mixing and transmission between individuals. A large number of species in the same roost might not only increase the rates of contact between bat populations, but also provide paths for cross-species diffusion and subsequent seeding events in other caves thanks to individual mobility. This suggests that infection cycles may be maintained among different host species, facilitating transient epidemics through extinction and recolonization events in a spatially structured environment, and overall potentially contributing to the spatial circulation of the virus at the regional scale.

Disease progression within individual hosts remains undefined, however it is expected to account for mechanisms to escape lethal infection and replenish the population of susceptible individuals to sustain transmission, contrasting the characteristic long lifespan of bats. Findings from passive and active surveillance suggest that bats may be capable of being exposed to lyssaviruses without dying^29^. No evidence has however addressed so far this aspect in a satisfactory or definitive way, mainly because of the scarce available knowledge on bat immunology^40^. Experimental infections in the primary host of EBLV-1 highlighted a strong dependence of the efficiency of transmission and probability of causing rabies disease on the virus transmission route^41^. The absence of virus-neutralizing activity in all sera under all transmission conditions is however in sharp contrast with field data from Spain, France, and Germany^25,30,33,35,42^. The presence of EBLV-1 neutralizing antibody response in healthy individuals appears to be relatively common and have been documented in *E. serotinus* and in other bat species including *M. myotis* and *M. schreibersii* in various regions^30,33,35,42,43^. While its interpretation remains rather difficult, previous work suggested this response may result from bats recovering from the infection following EBLV exposure^30^. Direct evidence of transmission during abortive or subclinical infection under natural conditions is indeed difficult to achieve with active surveillance as lyssaviruses are excreted only for short periods^25,30^. A recent longitudinal survey of *E. serotinus* colonies in France^42^ found for the first time viral RNA in bats saliva concomitant with virus excretion, and later followed by seropositivity, suggesting that transmission may occur during subclinical infection. In addition, individual waves of seroconversion and waning of immunity were reported in the same colony, similar to previous results obtained for *M. myotis* in Spain^44^.

Where knowledge gaps in bat ecology, epidemiology, and immunology hinder a comprehensive assessment of the mechanisms for EBLV-1 persistence in European bats, mathematical models provide a synthetic framework for hypotheses testing that can help improve our understanding of the spatial patterns reported by observational studies and identify important drivers for persistence. Such cross-disciplinary integrative modeling was previously suggested as a relevant research avenue to provide additional insights into the infectious disease dynamics with implications for our understanding of zoonotic disease emergence and associated risk for humans^2^. Here we develop a data-driven mechanistic metapopulation model for EBLV-1 spatial diffusion in the *Miniopterus schreibersii* and *Myotis myotis* non-synanthropic bat species in a system of caves in Catalonia, a region in the North-East of Spain. The model builds on existing data from a long-term field survey of EBLV-1 infection in natural bat colonies in the region^33,35,38^. *M. myotis* live as a single colony of few hundred individuals in the cave called Can Palomeres. *M. schreibersii* is a regional migratory species following a complex annual migration from cave to cave in the region. The two species share the same habitat in Can Palomeres during summer months. Through the use of spatially-resolved demographic and migration data, we explore several hypotheses regarding unknown epidemiological (transmission potential), immunological (lethal infection, immunity) and ecological aspects (cross-species mixing, seasonality in mixing, migratory behavior) to identify the mechanisms responsible for the reported EBLV-1 persistence in the two species. Given the current limitations of global surveillance for zoonotic diseases, focusing on the dynamics of bat infectious diseases and improving our understanding of the mechanisms driving their persistence may provide useful information to complement the available scarce resources to predict epizootics and potential risk for humans.

## Results

### EBLV-1 metapopulation model design

**Figure 1:**
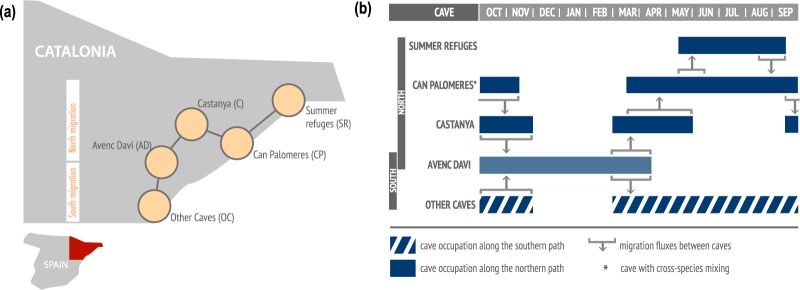
Schematic representation of the spatial model. (**a**) Schematic georeferenced diagram of the metapopulation model, composed of roosting caves (nodes) and migratory path (links) for *M. schreibersii* in the region of Catalunya. Can Palomeres is the cave where cross-species mixing may occur. **(b)** Temporal representation of the annual seasonal migration of *M. schreibersii*. Cave occupation is represented with filled rectangles (northern route) and striped ones (southern route).

We develop a multi-species metapopulation epidemic model^45–47^ where shelters occupied by bats are represented by patches and migration events between shelters are represented by links connecting different patches. After hibernation in Avenc Davi (AD), *M. schreibersii* population splits between northern and southern migration routes (Fig. 1a) for mating, birthing and breeding during spring and summer seasons, before reuniting itself in Avenc Davi at the start of the fall (Fig. 1b). *M. myotis* bats constitute a single colony located in Can Palomeres (CP) year-round where they may get in contact with *M. schreibersii* throughout spring/summer months.

**Figure 2:**
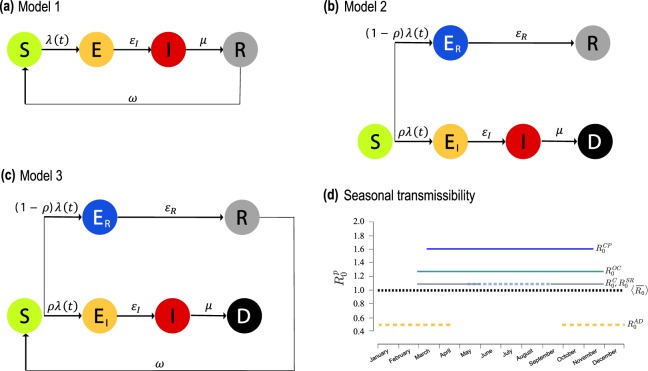
Disease progression models and seasonality of transmission. (**a**) Compartmental structure for model 1, where no infection-induced mortality is considered and immunity wanes with rate *ω*. *ε*_*I*_ is the rate of becoming infective following infection, and *μ* the recovery rate **(b)** Compartmental structure for model 2, considering lethal infection to occur with probability *ρ*, whereas non-lethally exposed individuals (*E*_*R*_) recover with rate *ε*_*R*_ to the permanently immune state. **(c)** As in (**b**) for model 3, where immunity wanes with rate *ω*. Demographic processes in the three diagrams are omitted for clarity. **(d)** Reproductive numbers ${R}_{0}^{p}$ for *M. schreibersii* along each patch *p* of the migration path. The values correspond to the maximum likelihood estimates. The average reproductive number of the metapopulation model, $\langle \bar{{R}_{0}}\rangle$, is also shown (black dashed curve).

Host mixing and possible local transmission of infection occurs within each populated patch. We propose three models for EBLV-1 infection dynamics in the bat species under study, to account for different hypotheses based on field observations and experimental knowledge. They are all based on a susceptible-exposed-infected-recovered (SEIR) compartmental scheme^48^, with variations to account for different immunological responses. Model 1 assumes non-lethal infection and loss of immunity (panel a of Fig. 2), as done in previous modeling works^44,49^. Model 2 considers the possibility for bats to develop a lethal infection (with a given probability $\rho =0.15,\,0.35,\,0.5$), alternative to a non-infectious state followed by permanent immunity (panel b). Model 3 is a variation of model 2 that considers temporary immunity of average duration *ω*^−1^ (panel c).

Seasonality characterizes migrations flows, hosts’ birth, and also transmission intensity, as the latter varies upon the degree of bats activity throughout the year. For *M. schreibersii*, we model it through a patch-dependent variation of the reproductive number *R*_0_ (Fig. 2d and Methods), a key epidemiological parameter measuring the average number of secondary cases that an infectious host can generate during the infectious period in a fully susceptible population^50^. For *M. myotis*, we consider a two-step function for *R*_0_ describing hibernation in winter months (as for *M. schreibersii* in Avenc Davì) and breeding and mating during the rest of the year (as for *M. schreibersii* in Can Palomeres).

Cross-species transmission between *M. schreibersii* and *M. myotis* may occur in Can Palomeres only where the two species share the habitat. We model it with a reduction in transmissibility, ${R}_{0}^{mix}=\alpha {R}_{0}^{CP}$ with $0\le \alpha \le 1$, to account for reduced mixing between different species (*α* = 0 refers to non-mixing conditions).

All parameters are described in Methods and Table S5 of the Supplementary Information.

### Predicted EBLV-1 persistence

We compute the persistence probability of EBLV-1 in each bat species as the fraction of stochastic simulations reaching the endemic equilibrium in both host populations, by varying the input reproductive number in Can Palomeres, $1.1\le {R}_{0}^{CP}\le 3.1$, and the *M. schreibersii* average immunity period, $180\le {\omega }^{-1}\le 780$. We compare numerical results across different models and hypotheses by introducing a metapopulation summary measure for *M. schreibersii* given by the average reproductive number of the metapopulation model across time and patches:

$$\langle \bar{{R}_{0}}\rangle =\frac{1}{365}\sum _{t}\,[\frac{{\sum }_{p}\,{R}_{0}^{p}{N}^{p}(t)}{{\sum }_{p}\,{N}^{p}(t)}],$$

where ${R}_{0}^{p}$ represents the reproductive number of patch *p*, and *N*^*p*^(*t*) indicates the *M. schreibersii* population size of patch *p* at time *t*.

**Figure 3:**
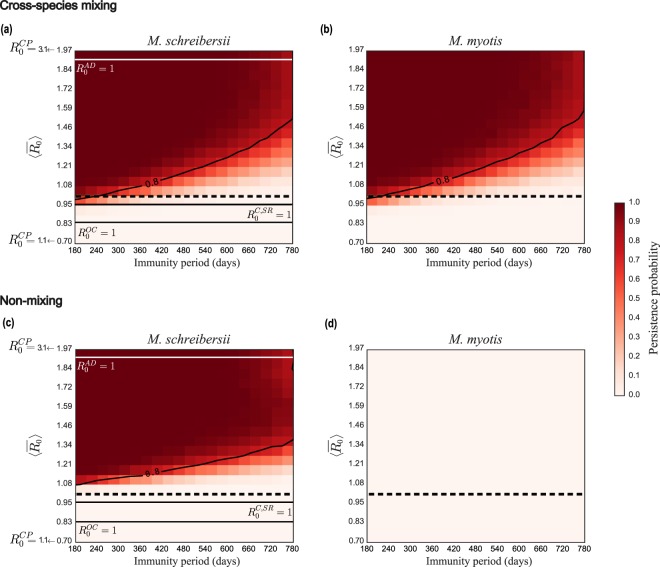
Persistence probability of EBLV-1 in *M. schreibersii* and in *M. myotis* bats in model 1. **(a,b)** Persistence probability for *M. schreibersii* (**a**) and for *M. myotis* (**b**) as a function of the average reproductive number of the metapopulation model $\langle \bar{{R}_{0}}\rangle$ and of the immunity period *ω*^−1^ in the mixing scenario. (**c,d**) as in (**a,b**) in the non-mixing conditions. Contour lines indicate a persistence probability of 80%. The dashed horizontal line refers to $\langle \bar{{R}_{0}}\rangle =1$. Solid horizontal lines refer to threshold conditions (${R}_{0}^{p}=1$) for the caves.

EBLV-1 circulation in both species is numerically recovered only in model 1 (temporary immunity and non-lethal infection) with cross-species mixing (Fig. 3, panels a and b). The absence of mixing between *M. schreibersii* and *M. myotis* or lethal infection lead instead to negligible or null probability of persistence in one of the species (*M. myotis*, Fig. 3, panel d) or both (Table S6 of the Supplementary Information, for different probabilities leading to the lethal infectious state), respectively. Also, density-dependent transmission would not allow persistence of the pathogen in any of the models explored (Table S6).

Persistence probability profiles are very similar in the two host populations in model 1 with cross-species mixing. Virus circulation is maintained for all values of the immunity periods explored, with high persistence ensured at lower transmissibilities if the immunity period is short. Mixing allows persistence also for values of the average metapopulation reproductive number close to or below the critical threshold, favoring viral circulation compared to the non-mixing case (Fig. 3, panels a and c).

We performed a sensitivity analysis to allow for variations in the ecological estimates (bat population sizes, Figs S4 and S5 of the Supplementary Information; starting date of migration events, Fig. S6; duration of migration events, Fig. S7), and in the assumed length of the infectious period (Fig. S3) yielding no variation in the predicted conditions for EBLV-1 circulation in both species.

### Maximum likelihood analysis

We use a maximum likelihood approach to compare the seroprevalence data from the two species^51^ with numerical results of model 1 and identify values of the reproductive number and immunity period mostly compatible with observations (see details in the Supplementary Information). Best estimate values for the unknown parameters are *ω*^−1^ = 390 (95% CI: 228–772) days and ${R}_{0}^{CP}=1.6$ (95% CI: 1.43–1.84). The latter is associated to a metapopulation average just above the critical threshold, $\langle \bar{{R}_{0}}\rangle =1.02$ (95% CI: 0.91–1.18), whereas the reproductive number is predicted to be largely subcritical in Avenc Davi during the hibernation period, ${R}_{0}^{AD}$ = 0.53 (95% CI: 0.48–0.61) (Table 1). With ${R}_{0}^{CP}$ set to its maximum likelihood estimate, we find the probability of viral persistence in *M. schreibersii* to vary strongly, decreasing from 82% to 1% when the immunity period varies within its 95% confidence interval, with a probability equal to 38% for the best estimate *ω*^−1^ = 390 days (Fig. S1 in the Supplementary Information). Such trend in the persistence probability is not substantially altered by variations in the assumed values for cross-species mixing (Fig. S2).

**Table 1:** Maximum likelihood estimates for the reproductive number.

Reproductive number	Maximum likelihood estimate and 95% CI
$\langle \bar{{R}_{0}}\rangle$	1.02 [0.91–1.18]
${R}_{0}^{CP}$	1.6 [1.43–1.84]
${R}_{0}^{OC}$	1.24 [1.11–1.43]
${R}_{0}^{C}$	1.06 [0.96–1.23]
${R}_{0}^{SR}$	1.06 [0.96–1.23]
${R}_{0}^{AD}$	0.53 [0.48–0.61]

### Experimental scenarios

To assess the impact of several ecological drivers on the probability of persistence of EBLV-1 in both species, we compare our data-driven metapopulation model with a set of experimental scenarios (see Methods).

**Figure 4:**
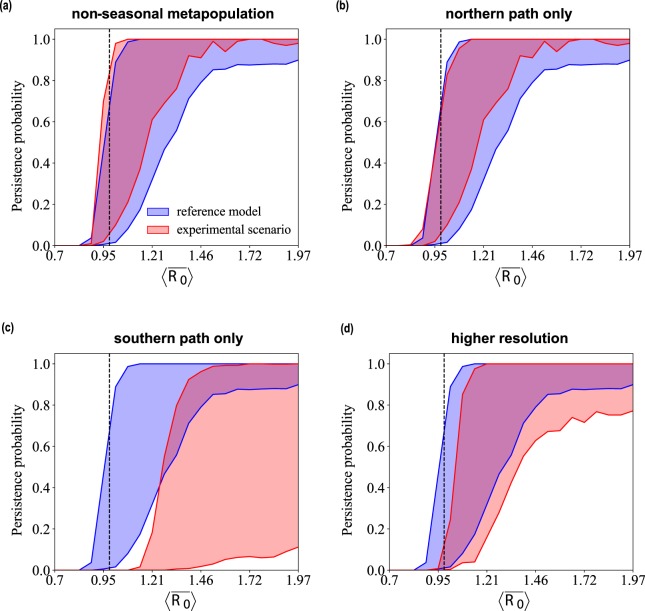
Comparison with experimental scenarios. Persistence probability for *M. schreibersii* as a function of the average reproductive number of the metapopulation model $\langle \bar{{R}_{0}}\rangle$ for values of the immunity period *ω*^−1^ spanning the estimated confidence interval. Each experimental scenario indicated in the plot title is compared with the reference model, corresponding to the data-driven metapopulation model. Numerical results are obtained for model 1 in mixing conditions. The dashed vertical line indicates $\langle \bar{{R}_{0}}\rangle =1$.

Discarding yearly seasonality of transmission and keeping a reproductive number constant in space and time leaves the persistence probability almost unaltered (*non-seasonal metapopulation*, Fig. 4). The essential role of migration is ensured by its northern portion, without which the likelihood of viral maintenance would be strongly reduced for a large set of values of $\langle \overline{{R}_{0}}\rangle$, becoming null when $\langle \overline{{R}_{0}}\rangle$ assumes its maximum likelihood estimate (*northern path only* vs. *southern path only*). Finally, considering the breakdown of the Summer refuges patch into smaller subpopulations, i.e. through a higher spatial resolution metapopulation model, would require a slight increase in transmissibility to reach the same persistence values of the reference model (*higher resolution*).

## Discussion

Through a spatially explicit multispecies metapopulation model based on available data from a long-term field survey on *M. schreibersii* and *M. myotis* bats in Spanish natural colonies, our study identified the main drivers for EBLV-1 persistence in the ecosystem under study and provide novel numerical evidence informing on previously unknown epidemiological, immunological and ecological factors.

Overall our findings indicate that EBLV-1 persistence relies on host spatial structure through the migration of *M. schreibersii* bats, on cross-species mixing with *M. myotis* population, and on a disease progression leading to survival of infected animals followed by temporary immunity. Even a low probability of developing lethal infection together with bats migration and reseeding through cross-species mixing would not be able to sustain the epidemic, regardless of the loss of immunity. While Lyssavirus infections are known to be generally lethal for mammals and for some bat species^52,53^, current knowledge from experimental and natural studies is not sufficient to accurately and satisfactorily define EBLV-1 disease progression in bats in natural conditions^30,33,35,40,42,43^. This is further complicated by biological and regulatory aspects. First, despite recent evidence for *E. serotinus* bats in France^42^, detecting a subclinical infective state in healthy bats in natural colonies is rather unlikely because of the short duration of the excretion period, and no such evidence exists yet for *M. schreibersii* or *M. myotis*. Second, the legal framework protecting European bats^54^ makes field studies particularly difficult to implement, as for instance marking bats is forbidden in Europe and special authorizations need to be requested to conduct these studies. Little but precious available field data are providing however an increasing body of evidence pointing to the possibility that various bat species may experience an infective subclinical state after being exposed to EBLV-1, followed by recovery and loss of immunity, with no associated increased mortality^25,30,35,42,44^, in agreement with our model results. A constant survival rate despite recurrent EBLV-1 epidemic cycles was reported for *M. myotis*^44^, supporting the findings of our model. Additional field data is needed to confirm our numerical predictions on EBLV-1 infection in *M. schreibersii*.

Two ecological factors emerge as critical drivers for viral persistence: cross-species mixing and host migration. We found that EBLV-1 circulation would not be maintained in a single colony alone in absence of mixing with other species. Multi-species colonies are indeed a phenomenon largely observed in the field that is known to favor virus exchange^33,55^. The importance of cross-species transmission of lyssaviruses was also reported in other ecological settings, identifying phylogenetic distance as the key determinant for cross-species transmission of rabies virus in North American bat species^11,56^. No study of this kind has been performed yet on EBLV-1 hosts species, and while the mixing intensity between *M. schreibersii* and *M. myotis* in Can Palomeres is unknown, our persistence estimates remain quite robust against this assumption. Our findings suggest that an increased public health attention should be focused on caves hosting multiple bat species with targeted surveillance and ecological fieldwork to improve our understanding of the role of species richness in viral exchange.

We find that transmission may not depend on host density in the cave. Many bat species are indeed known to form communities (families) that are stable over short terms (daily and nocturnal activities) and long terms (between migrations) thus including members of different generations, and whose sizes are independent on the colony size^57,58^. Given that virus transmission through bites and scratches^41^ requires close contact, the regularity of interactions with a limited number of hosts may explain the frequency-dependent transmission selected by our model. This result however seems to depend considerably on the species considered and the roosting behavior^59^. Additional modeling work focusing on different contexts might help better understanding such dependence across different conditions.

The other component critical for stable EBLV-1 circulation is the presence of the migratory species. Movements of infected individuals provide a mechanism for maintaining the chains of transmission through the seeding of epidemics in different patches. The importance of frequent immigration of infected hosts for persistence was also recognized in other settings^49,51,60,61^. Moreover, our findings indicate that the migratory species may contribute to pathogen persistence in species encountered along the migratory path. Given the potential for long-range seasonal movements of *M. schreibersii*^35,38^, this species may represent a central vector for spatial dispersion of EBLV-1 in southern Europe, where this bat species is abundant, possibly contributing to reconcile the discrepancy observed between the phylogeographical clusters of EBLV-1 variants and the geographic distribution of its common host species *E. serotinus*^36^.

In the ecological context under study, the northern portion of the migratory path is entirely responsible for viral persistence at the estimated immunity waning, likely because it is composed by a more complex spatial structure including a larger number of patches, thus creating more opportunities for seeding events sustaining coupled but non-synchronous patch epidemics^62^ that cannot be otherwise obtained with the southern path only. Increasing spatial resolution and resolving shelters sharing similar ecological and environmental conditions (such as the ones collectively grouped in Summer refuges) would not substantially alter our predictions. These findings are important to inform future field studies minimizing data collection efforts on roosts occupation.

Seasonality in mixing between hosts was instead found to have a negligible impact on EBLV-1 maintenance, suggesting that field efforts should be prioritized to provide an accurate characterization of the migration pattern, an important driver for EBLV-1 endemic circulation, instead of hosts’ degree of interaction.

The maximum likelihood analysis allows us to provide for the first time previously unidentified parameters characterizing the disease dynamics of EBLV-1 in *M. schreibersii*. We find that transmission among *M. schreibersii* bats in Can Palomeres (i.e. under the highest mixing conditions) is similar to what was previously estimated for *M. myotis* (*R*_0_ = 1.7) in natural colonies in Spain^44^. These conditions of transmissibility are associated to other caves being in close-to-critical or sub-critical conditions for efficient epidemic transmission. Persistence is thus mainly supported by transmission in Can Palomeres, though this cave only is not sufficient to maintain endemicity in *M. myotis*. The modeling predictions for the *M. schreibersii* immunity period are in the ballpark of previous empirical estimates of the maximum length of seropositive status observed in *M. myotis* (2–3 years)^35^ and in *E. serotinus* (4 years)^42^. Individuals are predicted to be immune on average for more than a year, thus hindering virus survival because of the slow replenishment of susceptible hosts. This effect is however counterbalanced by the migration of hosts that occurs on an annual timescale, providing opportunities for seeding events in naïve populations, a mechanism already identified by theoretical works to sustain viral circulation^62^. A rather large confidence interval is obtained for the estimate of the immunity period, indicating that the available serological data are not sufficient to significantly discriminate between approximately 1 year and 2 years of duration of immunity. Also, the likelihood of persistence is predicted to vary quite considerably within this range, as the system is found in the transition between null or negligible persistence and very high probability of viral maintenance. Additional cross-sectional studies in this colony and at higher temporal resolution may help improve our estimates.

The robustness of our model to a set of ecological, epidemiological, and immunological assumptions highlights that, despite the very limited knowledge of the system, our data-driven approach is able to clearly identify the mechanisms underpinning EBLV-1 circulation in the studied populations, contributing to the interpretation of field and laboratory data as well as providing important information for prioritizing and planning further empirical investigations.

Our study provides an example of how models can yield powerful insights for the identification of the main aspects of host ecology and interaction with the pathogen driving the maintenance of the infection in the host population. Though focused on a single ecological and epidemiological context, our model may be applied to other contexts of zoonotic interest through appropriate parameterizations, in order to derive insights into the infectious disease dynamics in bat populations and improve our understanding of the risk of transmission to domestic animals and humans. For instance, through a comparative approach across bat species and lyssavirus types, it may become possible to isolate host-pathogen specific drivers (e.g. the difference in pathogenicity between bats hosting rabies virus^59^ and EBLV-1) from commonalities (such as spatial structure and introductions) that would represent the key mechanisms for disease persistence across a range of lyssavirus systems. Understanding those, as well as the impact of possible anthropogenic changes on the infection dynamics in bats (e.g. a cave refuge for bats transformed into a touristic park), is essential for assessing bats capacity to serve as lyssavirus reservoirs in different geographic areas of the world and predicting the associated pathways for spillover events^9^.

Our study may be extended to other pathogens beyond lyssaviruses. While bats that host filoviruses like Ebola and Marburg viruses in Africa display very distinct ecological and behavioral traits compared to the European bat species studied here, recent surveillance work in Europe discovered a novel Ebolavirus-like filovirus in dead *M. schreibersii*^63^. Empirical evidence suggests that it may be pathogenic for the affected population, in contrast with the asymptomatic circulation of Ebola and Marburg viruses in fruit and insectivorous bats in Africa^64,65^. Given the large geographic distribution of *M. schreibersii*, and the critical role of its migratory behavior for pathogen persistence examined here, the discovery of a novel and potentially deadly filovirus in Western Europe is a significant concern. Integrative models may result to be particularly important to explore such scenarios where surveillance data is still rather poor, in order to strategize future empirical data collections.

With human-wildlife interactions still difficult to quantify^9^, considerable attention has shifted to animal populations. Spurred by outbreaks of high-impact viral zoonoses causing severe disease in human population, this paradigmatic shift has led to a remarkable increase in wildlife disease surveillance for early recognition of potential threats to humans (e.g. avian influenza in wild birds^66^, coronaviruses in bats^67–69^, etc.). Even after controlling for confounding factors such as investment in surveillance and research, bats were identified as carrying the highest proportion of zoonotic viruses across all mammals^6^. Moreover, hotspots of missing zoonoses were predicted for bats in Latin America and some parts of Asia, suggesting that pathogen diversity in bat species may be even larger than what currently known. Within such diversity, lyssaviruses still represent the only group of pathogens naturally circulating in bats for which a direct causal link between an infected bat and humans is well recorded^19,24,27,70–83^. Though the largest majority of rabid cases in humans is transmitted by rabid dogs, humans can develop rabies diseases after contact with a bat infected with a lyssavirus, leading to invariably fatal cases. Moreover, accurate human morbidity estimates for infections transmitted by bats are generally hard to achieve because standard diagnostic tests cannot readily resolve the causative lyssavirus^14^. Rabies vaccination is generally recommended in specific contexts to prevent and mitigate occupational hazards, often leading to a heavy financial burden on public health infrastructure^84^.

Our study is affected by some limitations. We did not consider *E. serotinus* bats in our model, the host species associated with the large majority of EBLV-1 cases detected in Europe^19,20,28^. While present in the region under study, its synanthropic behavior (i.e. living close to humans populations) likely precluded interactions with *M. schreibersii* and *M. myotis*, which instead clearly exhibit a non-synanthropic behaviour, roosting in natural caves and in abandoned mines^35^. Moreover, our model considers the two host populations to be closed and isolated in the region under study. While contacts with other non-synanthropic bat species of smaller population sizes may occur in Can Palomeres, our findings show that 2 host species are enough to self-sustain the epidemic given cross-species mixing and 1-species migration, with no need for additional introductions from other sites. This may appear to be in contrast with was found in other contexts, e.g. in a rabies virus model in vampire bats where persistence was largely dependent on immigration of infected individuals^51^. Our model however already accounts for seeding events from one patch to another thanks to the migration of *M. schreibersii*. Similarly to the study by Blackwood *et al*., we find indeed that the virus would not be maintained in a single population only.

The uncovered strong spatial component to transmission dynamics may help understanding the observed spatial diffusion of the virus at a larger scale on the continent and across a diverse range of host species, through long-range migration and seeding of local populations. Given the primary role of bats in carrying the largest proportion of zoonotic pathogens across mammals, determining how hosts enable persistence and which host species are critical in a multi-host infectious disease setting is essential for disease prevention and control. Our framework can readily be extended also to other zoonotic viral pathogens of public health concern circulating in spatially dispersed bat populations.

## Methods

### Migration

Following hibernation in Avenc Davì (AD), *M. schreibersii* population splits between northern and southern migration routes (Fig. 1b). On the northern route, *M. schreibersii* reach Castanya (C) for mating, and then they progressively start migrating to Can Palomeres (CP) for mating and birthing. An important fraction of the population (estimated 60%) continues the migration further north to other refuges composed of breeding or summer colonies (“Summer refuges”, SR, in Fig. 1a), where they stay approximately all summer. During the same period, the remaining bats stay in Can Palomeres where they share the refuge with *M. myotis*. Once summer is over, the entire *M. schreibersii* colony returns to Avenc Davì following the northern route in the opposite direction: from Summer refuges to Can Palomeres, to Castanya, to Avenc Daví for hibernation to conclude the annual migration. Bats following the southern route from Avenc Daví reach a set of caves near the coast (“Other caves”, OC) and return to Avenc Daví for hibernation, reuniting with the bats following the northern route. Migration rates are set to estimates from field data (Table S1 of the Supplementary Information)^38^, and a sensitivity analysis on starting date of migration events and on their duration was performed.

### EBLV-1 infection and vital dynamics

The three models considered are all a variation of a standard SEIR compartmental model, considering transmission in each patch and possible cross-species mixing in Can Palomeres. As an example, we provide here the force of infection for *M. schreibersii* in Can Palomeres in model 1, whereas full details for all models are reported in the Supplementary Information:

1 $$\lambda (t)={R}_{0}^{CP}\frac{({\varepsilon }_{I}+d)(\mu +d)}{{\varepsilon }_{I}}\frac{{I}_{s}(t)}{{N}_{s}(t)}+{R}_{0}^{mix}\frac{({\varepsilon }_{I}+d)(\mu +d)}{{\varepsilon }_{I}}\frac{{I}_{m}(t)}{{N}_{m}(t)},$$

where *ε*_*I*_ is the rate of becoming infective following infection, *μ* the recovery rate, *d* the mortality rate, *I*_*s*_(*t*) is the number of infective *M. schreibersii* bats in the Can Palomeres population of size *N*_*s*_(*t*) at time *t*, and analogously (${I}_{m}(t),\,\,{N}_{m}(t)$) for *M. myotis*.

We set disease progression parameters for *M. myotis* to available estimates from previous studies^44,49^. Lacking data characterizing the disease progression of EBLV-1 in *M. schreibersii*, we set the average durations of the incubation and infectious period to the values estimated for *M. myotis*, following previous modeling work^49^. Variations of these values are then considered for sensitivity analysis.

Seasonal birth is modeled during birthing summer season in Can Palomeres for both species, and natural death rate is assumed constant.

### Seasonality in EBLV-1 transmission in *M. schreibersii*

We consider the reproductive number ${R}_{0}^{CP}$ in Can Palomeres as an input to the model and corresponding to the highest transmissibility, associated with the highest mixing for mating and birthing occurring in that cave. Reproductive numbers in the other patches assume smaller values: ${R}_{0}^{AD}=\frac{1}{3}{R}_{0}^{CP}$ in Avenc Davì (i.e. the smallest value during hibernation period) and ${R}_{0}^{C}={R}_{0}^{SR}=\frac{2}{3}{R}_{0}^{CP}$ in Castanya and Summer refuges, based on expert opinion. For Other caves along the southern route we make the parsimonious hypothesis of ${R}_{0}^{OC}=\frac{{R}_{0}^{C}+{R}_{0}^{CP}+{R}_{0}^{SR}}{3}$, equal to the average value of northern’s path parameters.

### Numerical simulations and persistence analysis

We perform discrete stochastic numerical simulations of the EBLV-1 transmission in the host populations to account for the discrete nature of hosts and for stochastic extinction events that may be favored by small host population sizes. Time is considered to be discrete with a daily timescale. The epidemic is seeded with 100 infected *M. schreibersii* bats and 10 infected *M. myotis* bats in the hibernation period, and different initial conditions are explored for sensitivity. For each model and under each hypothesis considered, we ran 10^3^ stochastic simulations starting from the same initial conditions and reaching the endemic equilibrium. Simulations provide at each time step the number of *M. schreibersii* and *M. myotis* in each compartment in each cave, and the number of *M. schreibersii* migrating from one cave to another. The metapopulation framework is implemented in C++, and technical details for simulations are reported in the Supplementary Information.

### Experimental scenarios

To evaluate the role of seasonality in transmission, we build a *non-seasonal metapopulation* epidemic model with the same spatial structure of the data-driven metapopulation model (i.e. patches, demographics and migration dynamics of Fig. 1), but with no variation in the transmissibility associated to the caves. The reproductive number is assumed to be constant in time and space, and equal to $\langle \bar{{R}_{0}}\rangle$.

To identify the portions of the migration path that are mostly relevant for disease persistence, we consider a *northern path only* metapopulation model considering only bats following the path including Avenc Davi, Castanya, Can Palomeres and Summer refuges, and a *southern path only* metapopulation model considering instead only bats following the path from Avenc Davi to Other caves and return. Time and rates of migration events remain as in the full migration path.

To assess the role of spatial resolution in the identification of patches and associated mixing opportunities, we consider a *higher resolution* metapopulation model where all caves in the Summer refuges are independently considered as patches (corresponding migration flow estimates are provided in the Supplementary Information).

Finally, we test density-dependent transmission rates for EBLV-1 dynamics in both species, alternative to the frequency-dependent assumption considered in Eq. (1), to explore different mixing conditions.

## Electronic supplementary material

Supplementary Information file
